# Pretreatment of garden biomass using Fenton’s reagent: influence of Fe^2+^ and H_2_O_2_ concentrations on lignocellulose degradation

**DOI:** 10.1186/s40201-015-0167-1

**Published:** 2015-02-25

**Authors:** Vivek P Bhange, SPM Prince William, Abhinav Sharma, Jagdish Gabhane, Atul N Vaidya, Satish R Wate

**Affiliations:** Department of Biotechnology, Priyadarshini Institute of Engineering and Technology, Nagpur-19, Maharashtra, India; Solid and Hazardous Waste Management Division, National Environmental Engineering Research Institute, Nehru Marg, Nagpur-20, Maharashtra, India

**Keywords:** Fenton’s reagent, Pretreatment, Lignin, Cellulose, Garden biomass

## Abstract

Garden biomass (GB) is defined as low density and heterogeneous waste fraction of garden rubbish like grass clippings, pruning, flowers, branches, weeds; roots. GB is generally different from other types of biomass. GB is mostly generated through maintenance of green areas. GB can be processed for bio energy production as it contains considerably good amount of cellulose and hemicellulose. However, pretreatment is necessary to delignify and facilitate disruption of cellulosic moiety. The aim of the present investigation was to pretreat GB using Fenton’s reagent and to study the influence of Fe^2+^ and H_2_O_2_ concentrations on degradation of lignin and cellulose. The data were statistically analyzed using ANOVA and numerical point prediction tool of MINITAB RELEASE 14 to optimize different process variables such as temperature, concentration of Fe^2+^ and H_2_O_2_. The results of the present investigation showed that Fenton’s reagent was effective on GB, however, concentration of Fe^2+^ and H_2_O_2_ play crucial role in determining the efficiency of pretreatment. An increase in H_2_O_2_ concentration in Fenton’s reagent significantly increased the rate of cellulose and lignin degradation in contrast to increasing concentration of Fe^2+^ ion which led to a decrease in lignocellulosic degradation.

## Introduction

Biomass, in general, fourth largest energy source in the world, provides about 13% of world energy consumption [1]. Globally, biomass has an annual primary production of 220 billion oven dry ton [2]. Many cities, large or small, have developed gardens and recreational parks. The number of parks and other recreational centers, home gardens etc. contribute to the sizable quantum of garden biomass (GB) generation. Maintenance of green areas produces significant amount of waste in the form of GB [3]. GB is generally different from other types of biomass, and it is defined as low density and heterogeneous waste fraction of garden rubbish like grass clippings, pruning, flowers, branches, weeds, roots. The disposal of garden biomass is mainly through open burning, dumping and composting in India. Although these methods of disposal are universally applicable they neither recover energy nor eco-friendly except for composting.

GB contains recalcitrant or complex compounds such as cellulose and lignin, and relatively small amounts of saccharides, amino acids, proteins, aliphatic compounds and carbohydrates [3,4]. As GB is rich in cellulose, it can be used as a raw material for bio energy production after suitable pretreatment. Pretreatment is necessary to delignify and facilitate the disruption of lignocellulosic moiety. Pretreatment alters the structure of cellulose and making it more accessible to the enzyme that convert carbohydrate polymer into fermentable sugar [5,6].

There are different methods of pretreatment available for various substrates. However, it is necessary to evaluate every pretreatment process as the efficiency of pretreatment differs from substrate to substrate. Generally, pretreatment methods are either physical or chemical. Some methods incorporate both effects [7]. However, it is necessary to evaluate pretreatment processes for different substrates. Fenton’s reagent defined as a mixture of hydrogen peroxide and ferrous ion is one of the most effective methods for the oxidation of organic compounds.

Fenton process is a reaction between hydrogen peroxide (H_2_O_2_) and ferrous ion (Fe^2+^), producing the hydroxyl radical (•OH). •OH radical is a strong oxidant capable of oxidization and degradation of various organic compounds into carbon dioxide and water. Thus, the degradation process could be increased with increasing •OH concentration and vice versa [8-12].

$$ {\mathrm{Fe}}^{2+}+{\mathrm{H}}_2{\mathrm{O}}_2\to\ {\mathrm{Fe}}^{3+}+{\mathrm{O}\mathrm{H}}^{-}{+}^{\bullet}\mathrm{O}\mathrm{H} $$

The ferric ions produced during the reaction further react with hydrogen peroxide regenerating ferrous ions, thus continuing the process [13].

$$ {\mathrm{Fe}}^{3+} + {\mathrm{H}}_2{\mathrm{O}}_2\to {\mathrm{Fe}}^{2+} + \mathrm{H}\mathrm{O}\mathrm{O}\bullet + {\mathrm{H}}^{+} $$

However, the efficiency of Fenton’s reaction depends mainly on H_2_O_2_ concentration, Fe^2+^/H_2_O_2_ ratio, pH and reaction time [14].

In the present study we aimed at evaluating the effectiveness of Fenton’s reaction for pretreatment with a major emphasis on the influence of Fe^2+^ and H_2_O_2_ concentrations on degradation of lignin and cellulose.

## Materials and methods

### Preparation of the feedstock

Garden biomass (GB) consisting of grass cuttings, fallen leaves, flowers, roots, twigs etc. were collected from the garden area of National Environmental Engineering Research Institute (NEERI). After initial screening, GB was air-dried for24 hours followed by 3 days of sun drying. The dried material was pulverized using a pulveriser to the size of 1 to 5 mm for further experiments and stored in an air tight container.

### Pretreatment by Fenton’s reagent

Fenton’s reagent was prepared by mixing FeSO_4_.7H_2_O and H_2_O_2_ in distilled water in different proportions. The FeSO_4_.7H_2_O concentration varied from 250 ppm to 1000 ppm and H_2_O_2_ concentration varied from 1000 ppm to 10000 ppm. Every time Fenton’s reagent was prepared fresh and used in the experiment. All the experiments were carried out with 5 g of GB and 100 mL of Fenton’s reagent of different composition. All the reactions were initially carried out at 30°C and repeated at 50 and 80°C. The reaction was carried out in a shaker and reaction time was varied from 60 min to 180 min. The reaction mixture was filtered and then the treated GB was thoroughly washed and dried at 60°C for 2 days. The concentration of lignin and cellulose was estimated as described in section 2.3.

### Analytical methods

The dried sample of GB was ground to powder for chemical analysis. The organic carbon content of GB was estimated by combustion method according to Nelson & Sommers, 1982 [15]. Known quantity (mg) of substrate (GB) and its hydrolysed residue after pretreatment was taken and analyzed for cellulose by HNO_3_- ethanol method. Lignin content of samples was estimated by 72% (w/w) H_2_SO_4_ method and hemicellulose by Liu method [16]. The total nitrogen (TN) content of the sample was estimated using LECO Protein-Nitrogen Analyzer (Model FP528).

### Evaluation of cellulose and lignin degradation

Degradation of cellulose and lignin was evaluated on the basis of solid recovery [17,18] and actual degradation was calculated on the basis of residual concentration after pretreatment.

#### Cellulose recovery

Actual degradation (g) and actual degradation (%) of cellulose and solid recovery was calculated according to following formula:

$$ \begin{array}{c}\mathrm{Solid}\ \mathrm{r}\mathrm{ecovery}\ \left(\%\right)=\frac{\mathrm{Dry}\ \mathrm{weight}\ \mathrm{o}\mathrm{f}\ \mathrm{sample}\ \mathrm{after}\ \mathrm{pretreatment}}{\mathrm{Initial}\ \mathrm{weight}\ \mathrm{o}\mathrm{f}\ \mathrm{sample}\ \left(\mathrm{g}\right)} \times 100\\ {}\mathrm{Recovered}\ \mathrm{cellulose}\ \left(\mathrm{g}\right)=\frac{\mathrm{Conc}.\ \left(\%\right)\ \mathrm{o}\mathrm{f}\ \mathrm{cellulose}\ \mathrm{after}\ \mathrm{pretreatment} \times \mathrm{Solid}\ \mathrm{r}\mathrm{ecovery}}{\mathrm{Material}\ \mathrm{taken}\ \mathrm{f}\mathrm{o}\mathrm{r}\ \mathrm{pretreatment}}\end{array} $$

Actual degradation (g) and Actual degradation percentage (%) of cellulose was calculated by following formula:

$$ \begin{array}{c}\mathrm{Actual}\ \mathrm{degradation}\ \left(\mathrm{g}\right)\ \mathrm{of}\ \mathrm{cellulose} = \mathrm{Initial}\ \mathrm{conc}.\ \mathrm{of}\ \mathrm{cellulose}\ \hbox{--}\ \mathrm{Recovered}\ \mathrm{cellulose}\\ {}\mathrm{Actual}\ \mathrm{degradation}\ \left(\%\right)\ \mathrm{of}\ \mathrm{cellulose} = \frac{100 \times \mathrm{Actual}\ \mathrm{degradation}\ \left(\mathrm{g}\right)\ \mathrm{of}\ \mathrm{cellulose}}{\mathrm{Initial}\ \mathrm{conc}.\ \mathrm{of}\ \mathrm{cellulose}}\end{array} $$

#### Lignin recovery

Actual degradation (g), actual degradation (%) of lignin and solid recovery was calculated according to following formula:

$$ \begin{array}{c}\mathrm{Solid}\ \mathrm{r}\mathrm{ecovery}\ \left(\%\right)=\frac{\mathrm{Dry}\ \mathrm{weight}\ \mathrm{o}\mathrm{f}\ \mathrm{sample}\ \mathrm{after}\ \mathrm{pretreatment}}{\mathrm{Initial}\ \mathrm{weight}\ \mathrm{o}\mathrm{f}\ \mathrm{sample}\ \left(\mathrm{g}\right)} \times 100\\ {}\mathrm{Recovered}\ \mathrm{lignin}\ \left(\mathrm{g}\right)=\frac{\mathrm{Conc}.\ \left(\%\right)\ \mathrm{o}\mathrm{f}\ \mathrm{lignin}\ \mathrm{after}\ \mathrm{pretreatment} \times \mathrm{Solid}\ \mathrm{r}\mathrm{ecovery}}{\mathrm{Material}\ \mathrm{taken}\ \mathrm{f}\mathrm{o}\mathrm{r}\ \mathrm{pretreatment}}\end{array} $$

Actual degradation (g) and Actual degradation percentage (%) of lignin was calculated by following formula:

$$ \begin{array}{c}\mathrm{Actual}\ \mathrm{degradation}\ \left(\mathrm{g}\right)\ \mathrm{of}\ \mathrm{lignin} = \mathrm{Initial}\ \mathrm{conc}.\ \mathrm{of}\ \mathrm{cellulose}\ \hbox{--}\ \mathrm{Recovered}\ \mathrm{cellulose}\\ {}\mathrm{Actual}\ \mathrm{degradation}\ \left(\%\right)\ \mathrm{of}\ \mathrm{lignin}=\frac{100 \times \mathrm{Actual}\ \mathrm{degradation}\ \left(\mathrm{g}\right)\ \mathrm{of}\ \mathrm{lignin}}{\mathrm{Initial}\ \mathrm{conc}.\ \mathrm{of}\ \mathrm{lignin}}\end{array} $$

### Statistical guided experimental design and procedure

The Fenton’s pretreatment was statistically evaluated by applying statistical methodology viz. analysis of variance (ANOVA) followed by response surface methodology for process optimization [19,20]. The experimental runs were designed to cover variables that assess impact of pretreatment on cellulose and lignin degradation. The effects of Fe^2+^ concentration (X1), Hydrogen peroxide concentration (X2) and Temperature (X3) on lignin and cellulose degradation were described statistically. The regression analysis was performed to estimate the response function as a second-order polynomial:

1 $$ Y = {\beta}_0+{\displaystyle \sum_{i=1}^K}{\beta}_i{X}_i^2+{\displaystyle \sum_{i=1}^K}{\beta}_{ij}{X}_i^2+{\displaystyle \sum_{i=1}^{k-1}}\times {\displaystyle \sum_{j=2}^k}{\beta}_{ij}{X}_i{X}_j $$

Where *Y* is the predicted response, β_*i*_, β_*j*_, β_*ij*_ are coefficients estimated from regression, they represent the linear, quadratic and cross-products of *X*_*1*_*,X*_*2*_*,X*_*3*_ on response.

A statistical program package MINITAB RELEASE 14, was used for regression analysis of the data obtained and to estimate the coefficient of regression equation. The equations were validated by analysis of variance (ANOVA) analysis. The significance of each term in the equation is to estimate the goodness of fit in each case. Response surfaces were drawn to determine the individual and interactive effects of test variable on degradation of respective components.

## Results & discussion

### Initial characterization of GB

GB was analyzed to find out concentration of various constituents such as lignin, cellulose, hemicellulose, organic matter, organic carbon etc. (Table 1).

**Table 1:** **Initial characterization of garden biomass**

**Parameter**	**Concentration (%)**
Total organic matter	94.10
Organic carbon	49.12
Cellulose	38.54
Hemicellulose	26.24
Lignin	25.68
Nitrogen	1.65

A perusal of results showed that GB contained 94.10% of total organic matter, 49.12% of organic carbon, 38.54% of cellulose, 25.68% of lignin and 26.24% of hemicellulose. The total nitrogen content of GB was found to be 1.65%.

### Model fitting

The Levels of process variables, design of experiment along with experimental and predicted responses is given in Table 2, 3 and 4, respectively.

**Table 2:** **Levels of process variables in un-coded form for Fenton pre-treatment**

**Process variables**	**Levels of process variables**		
Fe^2+^ concentration ppm (X_1_)	250	500	1000
Hydrogen Peroxide concentration (ppm) (X_2_)	1000	5000	10000
Reaction temperature (°C) (X_3_)	30	50	80

**Table 3:** **Design matrix along with predicted and experimental values for cellulose degradation (%) by Fenton’s pretreatment**

**Runs**	**Fe** ^**2+**^ **(ppm)**	**H**_**2**_ **O**_**2**_ **(ppm)**	**Reaction temperature (°C)**	**Cellulose degradation (%)**	
				**Observed value**	**Predicted value**
	250	1000	30	26.433	24.591
	250	1000	50	27.337	28.535
	250	1000	80	20.000	19.662
	250	5000	30	30.767	30.552
	250	5000	50	31.340	34.495
	250	5000	80	27.000	25.622
	250	10000	30	43.067	41.901
	250	10000	50	47.230	45.844
	250	10000	80	35.000	36.971
	500	1000	30	13.367	16.098
	500	1000	50	*	*
	500	1000	80	10.000	11.168
	500	5000	30	24.267	22.058
	500	5000	50	*	*
	500	5000	80	20.000	17.129
	500	10000	30	31.267	33.408
	500	10000	50	40.790	37.351
	500	10000	80	26.000	28.478
	1000	1000	30	16.933	15.496
	1000	1000	50	17.487	19.440
	1000	1000	80	14.000	10.567
	1000	5000	30	19.467	21.457
	1000	5000	50	*	*
	1000	5000	80	15.000	16.527
	1000	10000	30	32.800	32.806
	1000	10000	50	38.230	36.749
	1000	10000	80	27.000	27.876

*Outliers removed.

**Table 4:** **Design matrix along with predicted and experimental values for lignin degradation (%) by Fenton’s pretreatment**

**Runs**	**Fe** ^**2+**^ **(ppm)**	**H**_**2**_ **O**_**2**_ **(ppm)**	**Reaction temperature (°C)**	**Lignin degradation (%)**	
				**Observed value**	**Predicted value**
1	250	1000	30	43.000	41.940
2	250	1000	50	52.000	52.634
3	250	1000	80	39.800	36.885
4	250	5000	30	46.500	45.032
5	250	5000	50	55.113	55.725
6	250	5000	80	39.340	39.976
7	250	10000	30	47.630	48.821
8	250	10000	50	57.390	59.515
9	250	10000	80	43.520	43.766
10	500	1000	30	33.580	36.155
11	500	1000	50	45.210	46.848
12	500	1000	80	28.660	31.099
13	500	5000	30	41.563	39.246
14	500	5000	50	48.560	49.940
15	500	5000	80	36.220	34.190
16	500	10000	30	42.900	43.036
17	500	10000	50	55.230	53.729
18	500	10000	80	40.300	37.980
19	1000	1000	30	35.940	32.680
20	1000	1000	50	44.317	43.374
21	1000	1000	80	26.733	27.625
22	1000	5000	30	33.580	35.772
23	1000	5000	50	47.437	46.466
24	1000	5000	80	28.750	30.716
25	1000	10000	30	37.550	39.562
26	1000	10000	50	53.230	50.255
27	1000	10000	80	33.420	34.506

Full quadratic multiple regression analysis of experimental data yielded the following regression equations for the degradation of cellulose and lignin achieved through Fenton’s pretreatment:

### Cellulose degradation

2 $$ {\mathrm{Y}}_1=16.7866-0.0667432*{X}_1+0.000970293*{X}_2+0.985852*{X}_3+0.0000436*{X}_1*{X}_1+0.0000000866*{X}_2*{X}_2-0.00985*{X}_3*{X}_3 $$

### Lignin degradation

3 $$ {\mathrm{Y}}_2=1.81315-0.0393363*{X}_1+0.000782833*{X}_2+2.23014*{X}_3+0.0000216*{X}_1*{X}_1 - 0.00000000166\ *{X}_2*{X}_2-0.02119*{X}_3*{X}_3 $$

Where *Y*_*1*_ is the % cellulose degradation achieved by Fenton’s pretreatment, *Y*_*2*_ is % lignin degradation by Fenton’s pretreatment, *X*_1_ is Fe^2+^ concentration, *X*_2_ and *X*_3_ are hydrogen peroxide concentration (ppm) and reaction temperature, respectively.

Tables 3 and 4 show degradation of cellulose and lignin at different concentrations of Fe^2+^ and H_2_O_2_ in Fenton reagent. A perusal of results indicated that Fenton’s reagent is effective on GB. The degradation of cellulose and lignin responded positively to the concentration of H_2_O_2_ and reaction temperature. Whereas increasing concentration of Fe^2+^decreased the rates of lignin and cellulose degradation. The best effective concentration (BEC) of Fe^2+^ and H_2_O_2_ was found to be 250 ppm and 10000 ppm, respectively at a temperature of 50°C. Though the degradation of lignin and cellulose was significant at this BEC, compared to the other conventional methods such as alkali or H_2_O_2_ oxidation tried on other lignocellulosic biomass, Fenton’s pretreatment pronounced only a low level of delignification [21,22]. However, there is no such report to our search which exclusively deals with the effects of Fenton’s reagent on lignin and cellulose degradation in GB. The cellulose reduction rates as observed (47.23%) in the present investigation are slightly higher than that of Liu and Cheng [23] who reported maximum of 20.28% removal of cellulose and 20.09% of lignin using acid pretreatment on herbal residue. However, Ayeni et al. [24] reported 17% lignin removal by alkaline peroxide assisted wet air oxidation with no loss of cellulose. The effect of H_2_O_2_ alone on wood waste was also studied by Ayeni et al. [24] who reported 11% lignin removal without loss of cellulose.

The regression coefficients values for Fenton pretreatment with respect to cellulose and lignin removal is close to one (R^2^ > 95%), indicating the aptness of second order polynomial in predicating the response in terms of the chosen independent values, moreover the predicted values were found to be in close agreement with the experimental results (Table 2). The adjusted R^2^ value (94.31% and 93.76% respectively) obtained by correcting the R^2^ value for sample size and number of terms for cellulose removal is indicative of high significance of the model. The ANOVA model for the degradation of lignin and cellulose is shown in Table 5.

**Table 5:** **Analysis of variance (ANOVA) of model parameters**

**Terms**	**Coefficient**	**F**	**P**
**Cellulose degradation (%)**			
Constant	16.7866		
Fe (X_1_)	−0.0667432	61.47	0.000
H_2_O_2_ (X_2_)	0.000970293	222.65	0.000
Reaction temperature (X_3_)	0.985852	19.48	0.000
Fe * Fe (X_1_* X_1_)	4.36930E-05	24.77	0.000
H_2_O_2_* H_2_O_2_ (X_2_ * X_2_)	8.66348E-08	2.55	0.129
Reaction temperature * Reaction temperature (X_3_ * X_3_)	−0.00985858	25.89	0.000
R-Sq = 95.79% R-Sq(pred) = 91.36% R-Sq(adj) = 94.31%			
**Lignin degradation (%)**			
Constant	1.81315		
Fe (X_1_)	−0.0393363	86.45	0.000
H_2_O_2_ (X_2_)	0.000782833	47.74	0.000
Reaction temperature (X_3_)	2.23014	25.77	0.000
Fe * Fe (X_1_* X_1_)	2.15921E-05	0.006	0.006
H_2_O_2_* H_2_O_2_ (X_2_ *X_2_)	−1.66049E-09	0.970	0.970
Reaction temperature * Reaction temperature (X_3_ * X_3_)	−0.0211932	0.000	0.000
R-Sq = 95.20% R-Sq(pred) = 91.26% R-Sq(adj) = 93.76%			

The ANOVA demonstrates that the model is more significant. This is evident from the calculated *F*-values 64 and 66 for effect of Fenton’s pretreatment on cellulose and lignin removal respectively (*P* = <0.05). The ANOVA results also Indicate that the coefficients for linear effects are significant (*P* = <0.01) for cellulose degradation and for lignin removal. The positive linear effect for H_2_O_2_ concentration and temperature indicate an increase in cellulose and lignin removal with increase in peroxide concentration and temperature in contrast to the observed negative linear effect for Fe^2+^ concentration. The concentration of Fe^2+^ ions present in the pretreatment solution should be in catalytic amounts as over dosage leads to adsorption on the substrate which may lead to subdued processing activity after treatment.

Many studies reported in literature have revealed that the use of a much higher concentration of Fe^2+^ could lead to the self-scavenging of •OH radical by Fe^2+^ and induce the decrease in degradation rates [25-27]. According to Neyens & Baeyens, 2003, when the amount of Fe^2+^employed exceeds that of H_2_O_2_, the treatment tends to have the effect of chemical coagulation. When the two amounts are reversed, the treatment tends to have the effect of chemical oxidation [28].

**Figure 1:**
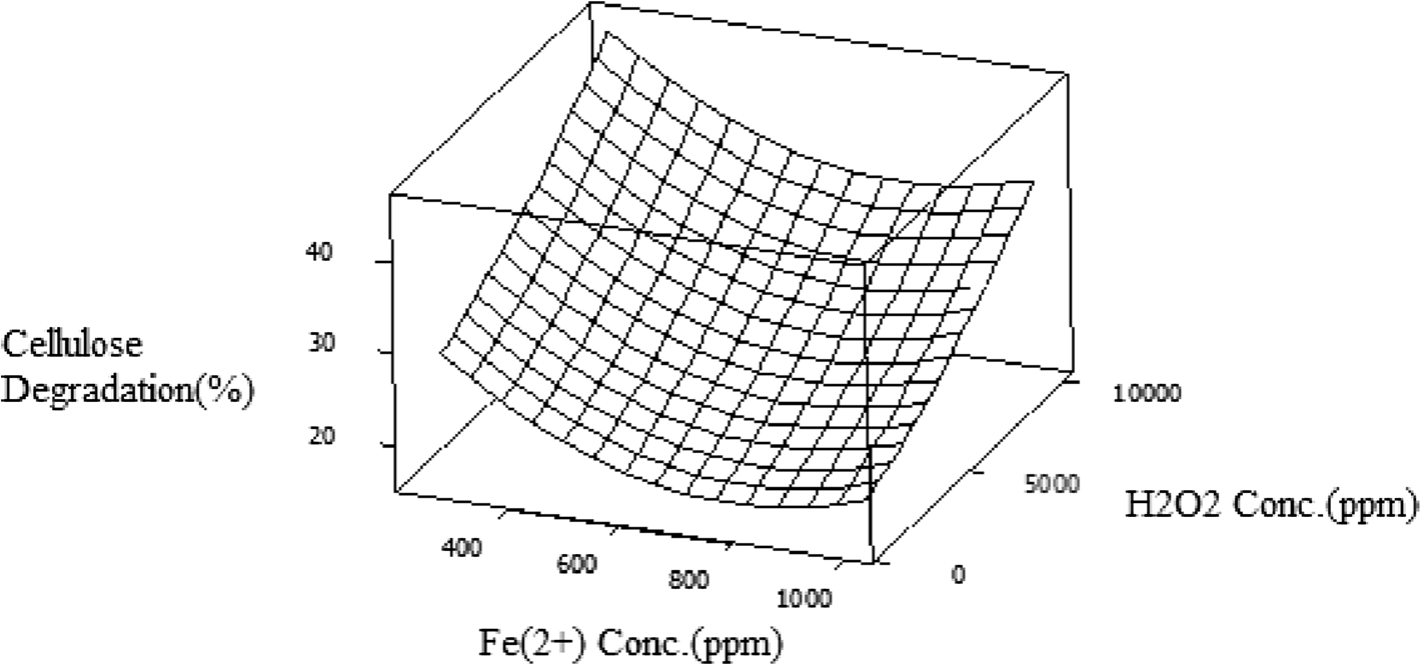
**Cellulose degradation (% w/w) as a function of Fe** ^**2+**^ **concentration (ppm) and H**_**2**_ **0**_**2**_ **concentration (ppm).**

**Figure 2:**
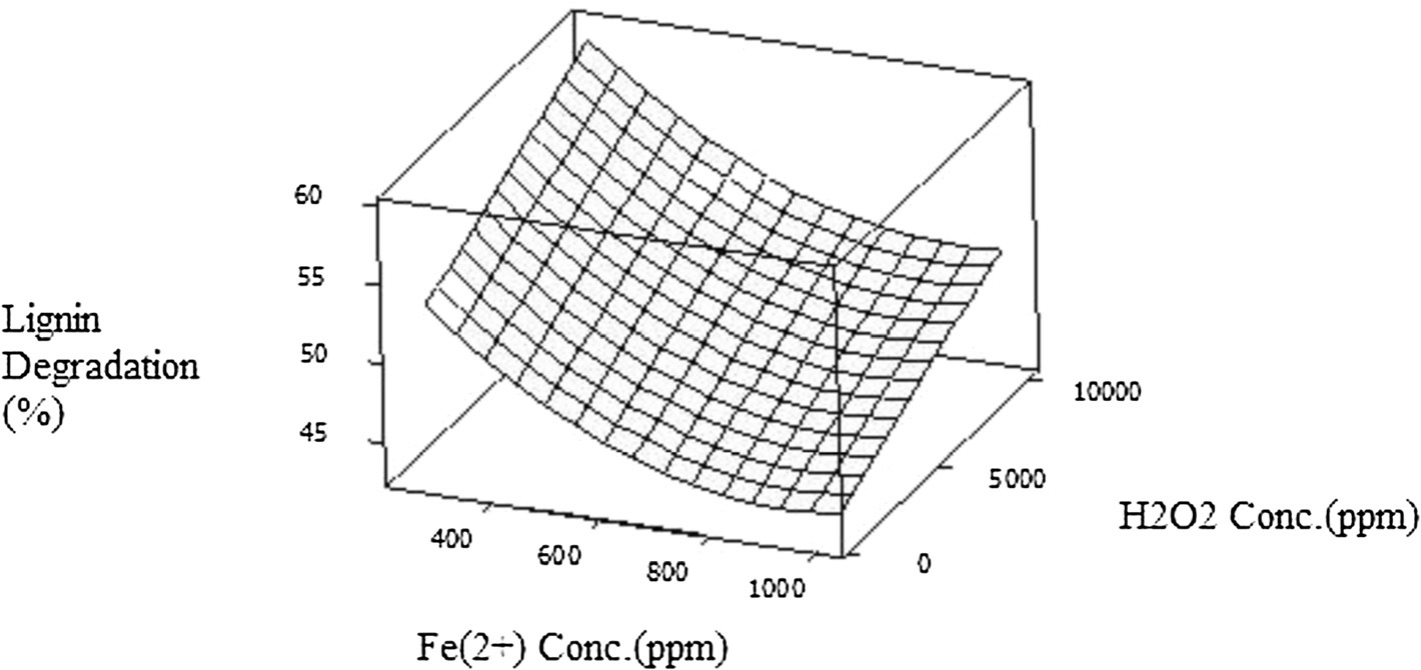
**Lignin degradation (% w/w) as a function of Fe2** ^**+**^ **concentration (ppm) and H**_**2**_ **0**_**2**_ **concentration (ppm).**

The effects of Fe^2+^ ion and H_2_O_2_ concentration on lignin and cellulose degradation when temperature was set at their centre point are shown in Figures 1 and 2. An increase in H_2_O_2_ concentration during pretreatment lead to considerable increase in lignin and cellulose degradation in contrast to increasing Fe^2+^ ion concentrations which lead to a decrease in cellulose removed from biomass. For example the cellulose and lignin removal increased from 13% to 31% & from 33% to 42% respectively at 500 ppm Fe^2+^ ion concentration when the H_2_O_2_ concentration was increased from 1000 to 10000 ppm.

The interactive effect of reaction time was however insignificant in Fenton pretreatment for lignin and cellulose removal and hence omitted from the regression analysis.

Overall, there is a predominance of the linear effects over the quadratic and interactive effects for both lignin and cellulose removal from the biomass. Higher peroxide concentrations lower Fe^2+^ concentrations higher reaction temperatures favour cellulose and lignin removal from the biomass.

## Conclusion

Effect of Fenton’s pretreatment on lignin and cellulose degradation of GB was studied. The results showed that Fenton’s reagent was effective on GB, however, concentration of Fe^2+^ and H_2_O_2_ play crucial role in determining the effectiveness of lignin and cellulose degradation. An increase in H_2_O_2_ concentration in Fenton’s reagent significantly increased the rates of cellulose and lignin degradation in contrast to increasing Fe^2+^ ion concentrations which led to a decrease in lignin and cellulose degradation. Further studies are necessary to compare and contrast Fenton’s pretreatment with other pretreatments and to understand the compatibility of Fenton’s pre-treated biomass for bioenergy production.

## References

[1] Hall DO, House J, Scrase I. Overview of biomass energy, in industrial uses of biomass Energy-The example of Brazil. In: Rosill Calle F, Bajay S, Rothman H, editors. Taylor & Francis, London;2000. p26.

[2] Hall DO, Rosill CF (1998). Biomass Resources other than wood.

[3] Tai H-S, He W-H (2007). A novel composting process for plant wastes in Taiwan military barracks. Resour Conserv Recycl.

[4] Cofie Olufunke. Adams Bradford A (2006). Organic Waste Reuse for Urban Agriculture.

[5] Bak JS, Ko JK, Han YH, Lee BC, Choi IG, Kim KH (2009). Improved enzymatic hydrolysis yield of rice straw using electron beam irradiation. J Ferment Technol.

[6] Mosier N, Wyman C, Dale B, Elander R, Lee YY, Holtzapple M (2005). Features of promising technologies for pretreatment of lignocellulosic biomass. Bioresour Technol.

[7] Hsu TA (1996). Pretreatment of Biomass, Handbook of Bioethanol, production and utilization.

[8] Lin SH, Lo CC (1997). Fenton process for treatment desizing wastewater. Water Res.

[9] Tang WZ, Huang CP (1996). 2,4-Dichlorophenol oxidation kinetics by Fenton’s reagent. Environ Technol.

[10] KangYW HKY (2000). Effect of reaction conditions on the oxidation efficiency in the Fenton process. Water Res.

[11] Hsueh CL, Huang YH, Wang CC, Chen CY (2005). Degradation of azo dyes using low iron concentration of Fenton and Fenton-like system. Chemosphere.

[12] Muruganandham M, Swaminathan M (2004). Decolourisation of reactive orange by Fenton and photo-Fenton oxidation technology. Dyes Pigments.

[13] Prateek J, Nadanathangam V (2012). Effect of Fenton’s pretreatment on cotton cellulosic substrates to enhance its enzymatic hydrolysis response. Bioresour Technol.

[14] Barbusiński K (2009). Fenton reaction-controversy concerning the chemistry. Ecol Chem Eng.

[15] Nelson DW, Sommers LE, Page AL (1982). Total carbon, organic carbon and organic matter. Methods of Soil Analysis Part II.

[16] Liu S (2004). Analysis and Measurement in Papermaking Industry.

[17] Sluiter A, Ruiz R, Scarlata C, Sluiter J, Templeton D (2008). Determination of Extractives in Biomass.

[18] Sluiter JB, Ruiz RO, Scarlata CJ, Sluiter AD, Templeton DW (2010). Compositional analysis of lignocellulosic feedstocks. 1. Review and description of methods. J Agric Food Chem.

[19] Montgomery DC, Anderson W, Russell S, Aiello E (2001). Design and Analysis of Experiments.

[20] Mathews P (2005). Design of experiments with MINITAB.

[21] Kumar P, Barrett DM, Delwiche MJ, Stroeve P (2009). Methods for pretreatment of lignocellulosic biomass for efficient hydrolysis and biofuel production. Ind Eng Chem Res.

[22] Mosier N, Hendrickson R, Ho N, Sedlak N, Ladish MR (2005). Optimization of pH controlled liquid hot water pretreatment of corn stover. Bioresour Technol.

[23] Liu C-Z, Cheng X-Y (2009). Microwave-assisted acid pretreatment for enhancing biogas production from herbal-extraction process residue. Energy Fuels.

[24] Ayeni AO, Hymore FK, Mudliar SN, Deshmukh SC, Satpute DB, Omoleye JA (2013). Hydrogen peroxide and lime based oxidative pretreatment of wood waste to enhance enzymatic hydrolysis for a biorefinery: Process parameters optimization using response surface methodology. Fuel.

[25] Chen R, Pignatello JJ (1997). Role of quinone intermediates as electron shuttles in Fenton and photo assisted Fenton oxidations of aromatic compounds. Environ Sci Technol.

[26] Joseph JM, Destaillats H, Hung HM, Hoffmann MR (2000). The sonochemical degradation of azobenzene and related azo dyes: rate enhancements via Fenton’s reactions. J Phys Chem A.

[27] Hameed BH, Lee TW (2009). Degradation of malachite green in aqueous solution by Fenton process. J Hazard Mater.

[28] Neyens E, Baeyens J (2003). A review of classic Fenton’s peroxidation as an advanced oxidation technique. J Hazard Mater.

